# Fear of Cancer Recurrence Before Treatment in Patients Newly Diagnosed With Breast Cancer: Cross-Sectional Study

**DOI:** 10.2196/89346

**Published:** 2026-04-10

**Authors:** Yun Ding, Huiting Zhang, Qiaoling Zhong, Lijuan Zhang, Huiying Qin

**Affiliations:** 1 State Key Laboratory of Oncology in South China, Guangdong Provincial Clinical Research Center for Cancer, Sun Yat-sen University Cancer Center, 651 Dongfeng East Road, Guangzhou, 510060, China, 86 02087343852

**Keywords:** fear of cancer recurrence, FCR, breast cancer, self-disclosure, social support

## Abstract

**Background:**

Fear of cancer recurrence (FCR) is a common psychological disorder among patients with cancer. In patients newly diagnosed with breast cancer, fear and concern about recurrence often influence treatment decision-making. However, the prevalence of FCR before treatment and its key risk factors—such as age, sleep quality, economic status, self-disclosure, and social support—remain unclear.

**Objective:**

This study aims to describe the prevalence and severity of FCR before treatment in patients newly diagnosed with breast cancer and analyze its predictive factors, thereby providing a scientific basis for interventions to reduce such fear.

**Methods:**

A cross-sectional study was conducted among patients newly diagnosed with breast cancer prior to treatment at a specialized oncology hospital in Guangdong Province, China, between February 2023 and July 2023. Sociodemographic data were collected, including age, gender, ethnicity, religious belief, education level, and marital status. Clinical data included pathological stage, tumor type, and family history of cancer. The Distress Disclosure Index, the Chinese version of the Medical Social Support Scale, and the short form of the Fear of Progression Questionnaire were used. Univariate analyses and bivariate correlations were conducted to explore relationships between variables. Multiple regression analysis was performed to identify predictors of FCR.

**Results:**

A total of 350 patients newly diagnosed with breast cancer were enrolled. Among the participants, 48.3% (n=169) experienced FCR before initiating treatment. The mean total score for FCR was 33.44 (SD 8.00). Age, sleep quality, financial burden, monthly household income per capita, family relationships, self-disclosure, and social support accounted for 33.5% (*R*²) of the variance in FCR.

**Conclusions:**

FCR prior to treatment is common and relatively severe among patients newly diagnosed with breast cancer. Health care professionals should implement targeted interventions to promote self-disclosure and strengthen social support networks to alleviate FCR, facilitate informed treatment decision-making, and improve patients’ psychological readiness for treatment. The findings of this study underscore the urgent need for early identification and management of FCR in patients newly diagnosed with breast cancer. High levels of pretreatment FCR may impair patients’ psychological well-being and influence treatment decision-making, potentially leading to suboptimal treatment choices. Health care professionals should implement targeted interventions that promote self-disclosure and strengthen social support networks to alleviate FCR. These measures can enhance patients’ psychological readiness for treatment, facilitate informed decision-making, and ultimately improve clinical outcomes.

## Introduction

According to the latest data released by the International Agency for Research on Cancer, breast cancer ranks as the second most common cancer globally, following lung cancer. In 2022, approximately 2.3 million new cases of breast cancer were reported worldwide, accounting for 11.7% of all newly diagnosed malignancies [1]. In recent years, significant advances have been made in the diagnosis and treatment of malignant tumors. However, the challenges of recurrence and metastasis remain unresolved. These continue to be formidable barriers that modern medicine has yet to overcome. Even patients with ductal carcinoma in situ of the breast face a recurrence risk ranging from 4% to 20% [2]. Fear of cancer recurrence (FCR) refers to the fear, worry, and anxiety experienced by patients regarding the potential return or progression of cancer [3]. This psychological state often begins at the time of cancer diagnosis and may persist for many years thereafter, affecting patients’ emotional well-being and behavioral responses [4]. A study by Lidia et al [5] revealed that approximately 64.5% of patients with breast cancer experience FCR prior to treatment. Pretreatment FCR is likely to influence patients’ treatment decisions. For example, a systematic review indicated that some Asian women opt for mastectomy due to concerns about recurrence and a desire to avoid further treatment. As a result, they may miss the opportunity for breast-conserving surgery [6]. A qualitative study also indicated that survivors of breast cancer carrying the breast cancer gene (BRCA1/2) mutations may undergo prophylactic mastectomy due to fears of recurrence [7]. Thus, recognizing and addressing pretreatment FCR in patients with breast cancer is essential to support optimal treatment choices.

However, current literature primarily focuses on the occurrence of FCR among patients with breast cancer during or after treatment. Findings indicate that approximately half of these patients experience moderate to high levels of FCR, which negatively impacts their quality of life, psychological well-being, and treatment adherence [5,7]. In contrast, limited research has been conducted on patients newly diagnosed with breast cancer prior to treatment. Only a few studies have reported the presence of FCR during the period between diagnosis and the initiation of treatment [5,7]. However, the severity of this fear and its associated factors remains insufficiently explored. Therefore, conducting in-depth research on FCR before treatment in patients newly diagnosed with breast cancer holds significant clinical importance. Such research not only addresses a critical gap in the existing literature but also provides a foundation for early intervention strategies.

**Figure 1.**
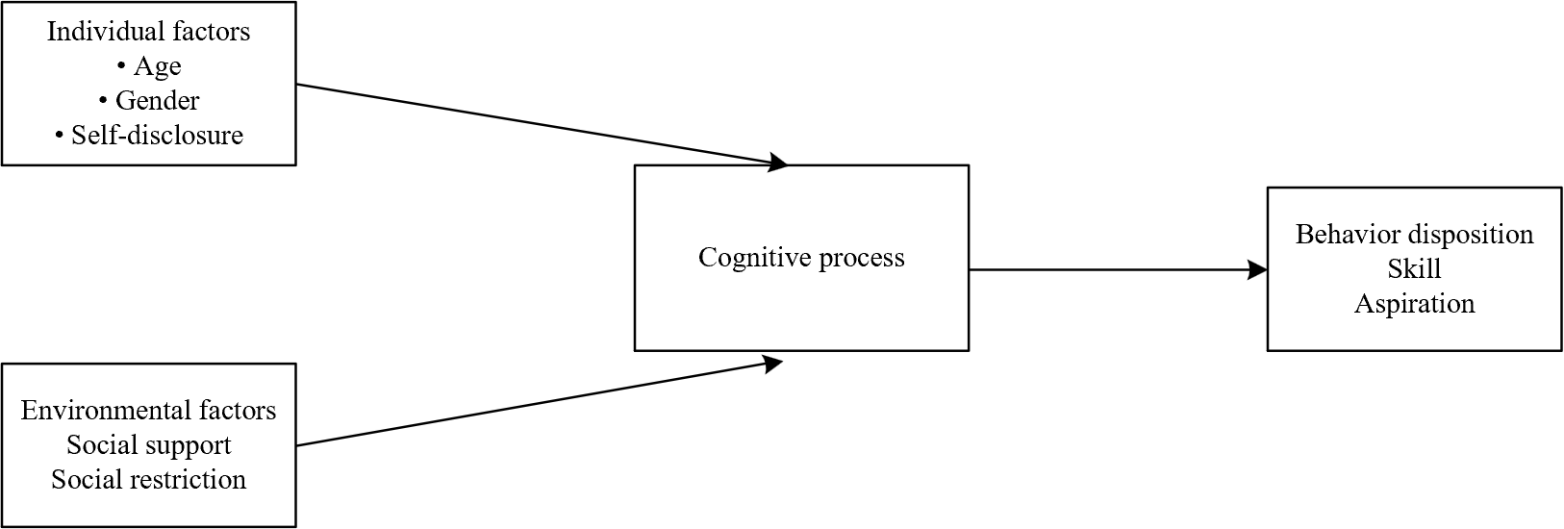
Framework of social cognitive theory.

According to social cognitive theory [8], human behavior and psychological outcomes are shaped by the dynamic interaction between personal factors, environmental influences, and cognitive processes. In the context of cancer diagnosis, personal factors such as self-disclosure and environmental factors such as social support may influence patients’ psychological adaptation through cognitive appraisal mechanisms (Figure 1). Self-disclosure, a core concept in psychology, refers to the process by which individuals share personal and private thoughts and emotions with others [9]. As a positive and effective form of communication, self-disclosure has been shown to negatively predict FCR [10]. Several studies have demonstrated that interventions promoting self-disclosure can enhance communication between patients with cancer and others, correct misconceptions regarding cancer recurrence, foster accurate illness beliefs, and ultimately reduce FCR. Specifically, self-disclosure allows patients to verbalize their fears and reorganize illness-related cognitions, which may reduce maladaptive interpretations of cancer recurrence risk [11-13]. Furthermore, social support has been identified as an independent predictor of FCR and plays a crucial role in alleviating it [14]. Low levels of social support are associated with increased FCR among patients with breast cancer, whereas high levels of support can encourage positive perceptions of health status and buffer the psychological distress caused by stressful events, thereby mitigating FCR [15,16]. At the same time, social support provides informational and emotional resources that help patients reinterpret threatening stimuli, correct misconceptions about recurrence, and strengthen perceived coping efficacy. Through these cognitive processes—such as illness perception, risk appraisal, and coping self-efficacy—patients may experience lower levels of FCR. Based on this theoretical framework, we hypothesize that higher levels of self-disclosure and stronger perceived social support are associated with lower levels of pretreatment FCR in patients newly diagnosed with breast cancer.

Patients newly diagnosed with breast cancer represent a unique population in which psychological factors play a pivotal role in treatment decision-making. Despite growing interest in the psychological dimensions of cancer care, no existing studies have empirically established the relationship between pretreatment FCR and self-disclosure or social support in this specific group. Given the potential influence of psychological readiness on treatment choices, it is crucial to explore whether targeted interventions that enhance self-disclosure and strengthen social support networks can effectively reduce FCR. Such interventions may ultimately empower patients to make more informed and appropriate treatment decisions. However, further research is needed to examine these associations and assess the clinical value of early psychosocial interventions in patients newly diagnosed with breast cancer.

Therefore, this study aims to describe the current status of FCR prior to treatment in patients newly diagnosed with breast cancer and to identify its risk factors based on social cognitive theory. The objectives of this study are as follows: (1) to explore the incidence and severity of pretreatment FCR in patients newly diagnosed with breast cancer; and (2) to identify the key risk factors contributing to pretreatment FCR in this population, thereby providing empirical evidence to inform the development of effective intervention strategies aimed at reducing FCR levels.

## Methods

### Study Design

This study used a cross-sectional design and was conducted from February 2023 to July 2023.

### Ethical Considerations

Ethical approval was obtained from the ethics committee of Sun Yat-sen University Cancer Center (B2023-700-01). The procedures used in this study adhere to the tenets of the Declaration of Helsinki, which emphasize respect for the dignity, autonomy, and privacy of research participants; the minimization of potential risks and the maximization of possible benefits; and the requirement for voluntary and informed consent prior to participation. Participants’ personal data were treated with strict confidentiality, and their rights and well-being were protected throughout the research process. Participants did not receive any compensation for their participation in this study.

### Participants

A convenience sampling method was used to recruit patients with breast cancer who sought medical care at a specialized oncology hospital in Guangdong Province, China, between February 2023 and July 2023. Inclusion criteria were as follows: (1) being aged 18 years or older; (2) first-time pathological diagnosis of stage 0-3 breast cancer; (3) no prior breast cancer–related treatment; and (4) an educational level of primary school or higher. Exclusion criteria included: (1) metastatic or recurrent breast cancer; (2) a history of other major organ diseases or malignancies; and (3) a diagnosis of psychiatric disorders or cognitive impairment. Written informed consent was obtained from all participants.

According to the empirical sample size calculation method based on regression analysis [17], the sample size should be 5 to 10 times the number of variables. The predictive factors included self-disclosure, social support, tumor stage, type, and a total of 23 influencing factors. Considering a 20% rate of invalid responses, the minimum sample size was calculated as 23×10/(1–0.20)=287.5 cases. Therefore, the minimum required sample size for this study was determined to be 288 cases.

### Measures

#### Sociodemographic and Clinical Characteristics

Sociodemographic data collected in this study included age, sex, ethnicity, religious beliefs, education level, marital status, number of children, average age of the youngest child, average age of all children, educational attainment, monthly household income per capita, financial burden, place of residence, living arrangement, family relationships, occupation prior to diagnosis, and method of medical expense payment. Disease-related variables included clinical stage, tumor type, comorbidities, family history of cancer, and sleep quality. Sleep quality was assessed using a single self-reported item in which participants rated their overall sleep status as “good,” “fair,” or “poor” during the period prior to treatment.

#### The Short Form of the Fear of Progression Questionnaire

The short form of the Fear of Progression Questionnaire was developed by German scholar Mehnert et al [18] in 2006 by simplifying the original Fear of Progression Questionnaire and is designed to assess the level of fear of disease progression in patients with cancer. In 2015, Wu Qiyun et al [19] translated and culturally adapted the scale into Chinese. The short form of the Fear of Progression Questionnaire consists of 12 items, categorized into 2 dimensions: physical health and social or family concerns. Each item is rated on a 5-point Likert scale, with all items scored positively. A total score of 34 or higher is commonly used to indicate a high level of fear, based on validation studies of the Chinese version of the scale [19]. The Cronbach α coefficient of the scale in this study was 0.883, indicating good internal consistency.

#### Revised Distress Disclosure Index

The Distress Disclosure Index was originally developed by Kahn and Hessling [20] to assess an individual’s tendency to disclose distressing experiences. In 2008, Chinese scholar Li Xinmin translated and revised the scale for use in the Chinese population to evaluate levels of self-disclosure [21]. The revised Chinese version consists of 12 items and uses a 5-point Likert scale ranging from 1 (“strongly disagree”) to 5 (“strongly agree”). Items 2, 4, 5, 8, 9, and 10 are reverse scored. The total score ranges from 12 to 60, with higher scores indicating a greater level of self-disclosure, while lower scores reflect a higher tendency toward concealment. The Cronbach α coefficient of the scale is 0.86, indicating good internal consistency.

#### Simplified Chinese Medical Outcomes Study Social Support Survey

The Simplified Chinese Medical Outcomes Study Social Support Survey (MOS-SSS-CM) was originally developed by Sherbourne et al [22] in 1991 to assess perceived social support among patients with chronic illnesses. In 2012, Li Huan [23] adapted the scale into a simplified Chinese version (MOS-SSS-CM) for use in the Chinese population. The total scale demonstrated good reliability, with a Cronbach α coefficient of 0.889 and a test-retest reliability of 0.77, indicating high internal consistency and stability. The scale contains 20 items across 4 dimensions: emotional or informational support, tangible support, affectionate support, and positive social interaction. Each item is rated using a 5-point Likert scale ranging from 1 to 5, based on the individual’s perceived frequency of support. The total score is converted to a 0-to-100 scale, with higher scores reflecting a higher level of perceived social support.

### Data Collection

For patients who met the inclusion and exclusion criteria, data collection was conducted after hospital admission and pathological diagnosis, but prior to the initiation of treatment. Upon obtaining written informed consent, a professionally trained research staff member distributed the questionnaires and guided participants in their completion. After completion, the researcher checked each questionnaire for completeness. Prior to the start of the study, this staff member received comprehensive training on research ethics, questionnaire content, completion guidelines, and communication skills with patients, ensuring effective interaction and the provision of appropriate support throughout the process.

To ensure data quality and minimize potential bias, standardized procedures were implemented during questionnaire administration. The research staff members received structured training on research ethics, questionnaire content, standardized instructions, and communication skills prior to data collection. All questionnaires were completed independently by participants, and responses were checked for completeness without influencing participants’ answers. Specifically, the researcher responsible for questionnaire administration was not provided with information about the patients’ treatment plans or clinical conditions during data collection, thus minimizing potential bias in the data collection process.

### Data Analysis

Data analysis was conducted by an independent researcher with extensive experience in clinical research and professional training in oncology and statistics. The dataset was deidentified prior to analysis to ensure confidentiality and objectivity.

All statistical analyses were performed using SPSS version 25.0 (IBM Corp), with a significance level set at *α*=.05; *P* values less than .05 were considered statistically significant. Descriptive statistics were used to characterize participants’ sociodemographic and clinical characteristics. Univariate analyses or bivariate correlations were initially conducted to identify potential factors associated with levels of FCR. ANOVA was used to examine differences in FCR scores across various sociodemographic and clinical characteristics (eg, education level, marital status, employment status, and household income). Pearson correlation analysis was performed to assess the relationships among self-disclosure, social support, and FCR in patients with breast cancer.

Variables demonstrating statistical significance in univariate analyses were subsequently entered into a multivariate regression model. A multiple linear regression analysis was conducted to identify independent predictors of FCR. The squared correlation coefficient (*R*²) was used to indicate the proportion of variance in the dependent variable that could be explained by the independent variables. A *P* value of 0.05 was considered the threshold for statistical significance.

## Results

### Analysis of Demographic Characteristics, Self-Disclosure, Social Support, and FCR Among Patients Newly Diagnosed With Breast Cancer Prior to Treatment

A total of 362 patients met the inclusion criteria and provided informed consent. As 12 patients refused to participate in the study, 350 patients finally completed the questionnaire, resulting in a loss rate of 3.31%. The descriptive data on sociodemographic and clinical characteristics, as well as Distress Disclosure Index and MOS-SSS-CM scores, are summarized in Table 1. A total of 350 patients with breast cancer, aged between 25 and 77 years (mean age: 45.39, SD 9.68), were included in the study, with 99.1% (n=347) being female. The majority reported a per capita monthly household income above 5000 RMB (US $689.22). Financial concerns were prevalent, with 63.7% of patients experiencing some degree of economic burden and 15.7% (n=55) reporting severe financial stress. Additionally, 55.7% (n=195) rated their sleep quality as average. The mean (SD) scores for self-disclosure and social support were 38.86 (9.31) and 70.57 (14.04), respectively. The mean FCR score among patients with breast cancer was 33.43 (SD 8.00), with the physical health dimension (18.11, SD 4.12) scoring higher than the social or family dimension (15.32, SD 4.66). Notably, 48.3% (n=169) of patients reported experiencing fear of recurrence prior to the initiation of treatment.

**Table 1.** Correlation of the short form of the Fear of Progression Questionnaire (FoP-Q-SF) with sociodemographic characteristics, clinical characteristics, the Distress Disclosure Index (DDI), and the Simplified Chinese Medical Outcomes Study Social Support Survey (MOS-SSS-CM; N=350).

Variables	Value	FoP^a^ score, mean (SD)	Statistics	*P* value
Age (y), mean (SD)	45.39 (9.68)	—^b^	10.99 (2, 347)^c^	<.001
Young adults (18-40), n (%)	121 (34.6)	35.34 (7.41)		
Middle-aged adults (41-60), n (%)	202 (57.7)	33.05 (8.06)		
Older adults (61-77), n (%)	27 (7.7)	27.78 (7.21)		
Gender, n (%)			–1.914 (348)^d^	.06
Men	3 (0.9)	24.67 (6.02)		
Women	347 (99.1)	33.51 (7.98)		
Ethnicity, n (%)			–0.192 (348)^d^	.85
Han ethnicity	338 (96.6)	33.0 (10.46)		
Minority	12 (3.4)	33.45 (7.91)		
Faith, n (%)			0.596 (348)^d^	.55
No	330 (94.3)	33.50 (7.97)		
Yes	20 (5.7)	32.40 (8.61)		
Marital status, n (%)			0.178 (2, 347)^c^	.84
Unmarried	17 (4.9)	33.65 (8.90)		
Divorced or widowed	19 (5.4)	34.47 (8.43)		
Married	314 (89.7)	33.36 (7.94)		
Number of children, n (%)			0.491 (3, 346)^c^	.69
Nulliparous	29 (8.3)	32.66 (8.24)		
1	140 (40)	33.05 (7.65)		
2	145 (41.1)	33.68 (8.23)		
≥3	37 (10.6)	34.58 (8.31)		
Average age of the youngest child (y), n (%)			2.487 (348)^d^	.01
18	222 (60.1)	34.24 (7.94)		
≥18	128 (36.6)	32.05 (7.94)		
Average age of all children (y), n (%)			2.433 (348)^d^	.02
<18	206 (55.1)	34.30 (7.89)		
≥18	144 (41.1)	32.20 (8.01)		
Educational level, n (%)			1.866 (2, 347)^c^	.16
Junior high school or lower	96 (27.4)	34.77 (7.73)		
High school or technical secondary school	84 (24)	33.07 (9.39)		
College degree or higher	170 (48.6)	32.86 (7.33)		
Monthly per capita household income (RMB^e^), n (%)			3.695 (2, 347)^c^	.03
≤3000	48 (13.7)	33.56 (8.30)		
3001-5000	111 (31.7)	35.05 (8.55)		
>5000	191 (54.6)	32.47 (7.46)		
Financial burden, n (%)			20.624 (2, 347)^c^	<.001
No financial burden	72 (20.6)	29.64 (7.26)		
Moderate financial burden	223 (63.7)	33.45 (7.56)		
Severe financial burden	55 (15.7)	38.36 (8.08)		
Place of residence, n (%)			3.442 (2, 347)^c^	.03
Rural area	81 (23.1)	35.26 (8.29)		
Town area	88 (25.1)	33.69 (7.37)		
Urban area	181 (51.8)	32.50 (8.05)		
Living arrangement, n (%)			0.863 (2, 347)^c^	.42
Living alone	20 (5.7)	35.60 (8.83)		
Living with friends	5 (1.4)	34.80 (11.16)		
Living with family	325 (92.9)	33.28 (7.90)		
Family relationship, n (%)			4.666 )（348）^d^	<.001
Nonharmonious	55 (15.7)	37.9 (7.55)		
Harmonious	295 (84.3)	32.60 (7.81)		
Occupation before diagnosis, n (%)			4.820 (2, 347)^c^	.01
Retired	52 (14.9)	30.46 (8.27)		
Unemployed	62 (17.7)	34.85 (7.81)		
Employed	236 (67.4)	33.72 (7.86)		
Type of health care payment, n (%)			0.488 (2, 347)^c^	.61
Self-paid	7 (2)	36.0 (6.21)		
Medical insurance	335 (95.7)	33.72 (7.97)		
Government-funded health care	8 (2.3)	32.0 (10.66)		
Pathological staging, n (%)			1.428 (3, 346)^c^	.23
0	31 (8.9)	34.29 (10.95)		
I	93 (26.6)	32.34 (8.02)		
II	157 (44.9)	34.24 (6.54)		
III	69 (19.7)	32.70 (9.28)		
Tumor type, n (%)			0.582 (348)^d^	.56
Noninvasive carcinoma	37 (10.6)	34.16 (10.21)		
Invasive carcinoma	313 (89.4)	33.35 (7.71)		
Family history of cancer, n (%)			–0.381 (348)^d^	.70
No	251 (71.7)	33.33 (8.00)		
Yes	99 (28.3)	33.70 (8.03)		
Comorbidity, n (%)			0.599 (348)^d^	.55
No	283 (80.9)	33.56 (7.87)		
Yes	67 (19.1)	32.91 (8.57)		
Sleep status, n (%)			9.399 (2, 347)^c^	<.001
Poor	67 (34.6)	35.04 (8.83)		
Fair	195 (34.6)	34.28 (7.34)		
Good	88 (34.6)	30.35 (8.01)		
Self-disclosure score, mean (SD)	38.86 (9.31)	—	–0.377^f^	<.001
Social support score, mean (SD)	70.57 (14.04)	—	–0.393^f^	<.001

a FoP: Fear of Progression.

b Not applicable.

c Means comparing the FoP of different characteristics with an ANOVA (*F* test).

d Means comparing the FoP of different characteristics with the *t *test. All *t* tests were 2-tailed.

e Currency conversion was based on the exchange rate on July 1, 2023 (1 RMB = US $0.137844).

f Means Pearson correlation with FoP-Q-SF.

### Analysis of Demographic Characteristics, Self-Disclosure, Social Support, and Their Correlations With FCR in Patients Newly Diagnosed With Breast Cancer Before Treatment

The results of the ANOVA indicated that there were statistically significant differences in FCR related to age, the average age of the youngest child, the average age of all children, monthly per capita household income, financial burden, place of residence, family relationships, occupation before diagnosis, and sleep status (*P*<.05). The Pearson correlation analysis revealed that both self-disclosure (*r*=−0.394; *P*<.01) and social support (*r*=−0.393; *P*<.01) were significantly and negatively associated with fear of cancer recurrence (Table 1).

### Multiple Regression Analysis of FCR in Patients Newly Diagnosed With Breast Cancer

A standard enter method linear regression analysis was performed, with FCR set as the dependent variable. Independent variables were those found to be statistically significant (*P*<.05) in univariate and correlation analyses, including patient age, average age of the youngest child, average age of all children, monthly per capita household income, financial burden, place of residence, family relationship, occupation before diagnosis, sleep quality, self-disclosure, and social support. A significant regression equation (*F*_11,337_=15.42; *P*<.001) was found, with an *R^2^* of 0.335 (Table 2). Younger age, poorer sleep quality, greater financial burden, lower self-disclosure, and lower social support were independently associated with higher FCR levels. Lower household income showed a trend toward association with higher FCR (*P*=.05).

**Table 2.** Multiple linear regression analysis of fear of cancer recurrence in patients with breast cancer(N=350).

Variables	B^a^	SE *B*	β^b^	*t *test (*df*)^c^	*P* value	*R* ^2^	95% CI
Constant	43.05	2.93		14.70 (337)	<.001	0.335	37.30 to 48.82
Age (y)							
Older adults (61-77)	—^d^	—^d^	—^d^	—^d^	—^d^	—^d^	—^d^
Young adults (18-40)	6.00	1.50	0.358	4.01 (337)	<.001	—^d^	3.06 to 8.94
Middle-aged adults (41-60)	3.76	1.42	0.232	2.65 (337)	.008	—^d^	0.97 to 6.54
Sleep status							
Good	—^d^	—^d^	—^d^	—^d^	—^d^	—^d^	—^d^
Fair	3.09	0.88	0.192	3.52 (337)	<.001	—^d^	1.36 to 4.82
Poor	2.68	1.15	0.132	2.32 (337)	.02	—^d^	0.42 to 4.95
Monthly per capita household income (RMB^e^)							
High income (>5000)	—^d^	—^d^	—^d^	—^d^	—^d^	—^d^	—^d^
Middle income (3001-5000)	0.19	0.86	0.011	0.22 (337)	.83	—^d^	−1.51 to 1.90
Low income (≤3000)	−2.33	1.21	−0.100	−1.94 (337)	.054	—^d^	−4.71 to 0.04
Financial burden							
No financial burden	—^d^	—^d^	—^d^	—^d^	—^d^	—^d^	—^d^
Severe financial burden	4.52	1.42	0.206	3.18 (337)	.002	—^d^	1.73 to 7.31
Moderate financial burden	2.35	0.96	0.141	2.44 (337)	.02	—^d^	0.45 to 4.24
Family relationship	−1.97	1.06	−0.09	−1.85 (337)	.07	—^d^	−4.06 to 0.12
Self-disclosure	−0.25	0.04	−0.286	−5.77 (337)	<.001	—^d^	−0.33 to -0.16
Social support	−0.10	0.03	−0.17	−3.15 (337)	.002	—^d^	−0.16 to -0.04

a B: unstandardized regression coefficient.

b β: standardized regression coefficient.

c All *t* tests were 2-tailed.

d Not applicable.

e Currency conversion was based on the exchange rate on July 1, 2023 (1 RMB = US $0.137844).

## Discussion

Patients newly diagnosed with breast cancer represent a unique population, in which psychological factors play a critical role in treatment decision-making. To our knowledge, this study is the first to investigate the relationships between sociodemographic and clinical characteristics, self-disclosure, social support, and FCR prior to treatment, and to identify significant predictors of FCR in this population.

### FCR Before Treatment in Patients Newly Diagnosed With Breast Cancer

This study found that FCR was a prevalent psychological issue among patients newly diagnosed with breast cancer prior to treatment, with approximately 50% experiencing clinically significant levels of fear. This proportion was lower than that reported by Han et al [24] but higher than that found by Li et al [25], suggesting heterogeneity in FCR across different patient groups. The discrepancy may be due to differences in study populations and timing of assessment. Han et al [24] focused on patients undergoing chemotherapy, during which FCR may be intensified by side effects and prolonged stress (including financial, occupational, and familial stressors). In contrast, the participants in this study were at the pretreatment stage, not yet exposed to treatment-related stressors, which may explain their relatively lower FCR. Li et al’s [25] study mainly involved postoperative patients. In our study, patients were newly diagnosed, facing difficulty accepting the diagnosis and perceiving uncertainty about future treatment, potentially contributing to high FCR. These findings highlight the importance of early psychological intervention for newly diagnosed patients.

### Younger Patients, Poor Sleep Quality, Low Income, and Financial Burden Exhibit Higher Levels of FCR

This study identified age as a significant factor influencing FCR in patients with breast cancer (*P*<.001), consistent with the findings of Zhang et al [26]. Younger patients tend to be more concerned with body image, and treatment-related changes, such as hair loss from surgery or chemotherapy, may trigger feelings of inferiority or depression, exacerbating FCR.

Sleep quality was significantly associated with FCR in the multivariate analysis, with poorer self-reported sleep linked to higher levels of fear [27]. However, as sleep quality was assessed using a single self-reported item and the study used a cross-sectional design, the directionality of this association cannot be determined. It is possible that sleep disturbances and FCR are interrelated through shared psychological mechanisms, such as heightened cognitive arousal or stress sensitivity. These findings suggest that sleep quality may be an important factor to consider when assessing psychological vulnerability in newly diagnosed patients. Future longitudinal studies using standardized sleep measures (eg, the Pittsburgh Sleep Quality Index) are needed to further clarify the nature and direction of this relationship.

Both univariate and multivariate analyses suggested that lower household income tended to be associated with higher levels of FCR; however, this association did not reach conventional statistical significance in the multivariate model (*P*=.054). In contrast, perceived financial burden remained a significant independent predictor of FCR, with patients reporting moderate or severe financial burden exhibiting higher FCR levels. These findings are consistent with the results reported by Niu et al [14]. Possible explanations include the substantial cost of cancer treatment and the additional financial concerns related to potential recurrence. Patients facing greater economic strain may experience heightened anxiety and uncertainty about future treatment expenses, which could amplify fear of recurrence. Notably, patients with medical insurance reported lower levels of FCR compared with those who paid out-of-pocket, suggesting that financial protection mechanisms may play a buffering role in psychological adaptation. Given that patients with breast cancer often require costly adjuvant therapies such as chemotherapy or radiotherapy, limited reimbursement for anticancer medications may exacerbate financial stress among economically vulnerable patients. Therefore, health care providers should assess patients’ financial situations when formulating treatment plans, offer cost-effective therapeutic options when feasible, and provide targeted psychological support. At the policy level, further strengthening the medical insurance system and expanding coverage for major illnesses may help alleviate financial burden and indirectly reduce FCR.

### Lower Levels of Self-Disclosure Are Associated With Higher FCR

This study found a significant negative correlation between self-disclosure and FCR. Self-disclosure, typically expressed through verbal communication of one’s thoughts and emotions, serves both as a personal coping mechanism and a psychological intervention strategy. It has been shown to alleviate psychological distress and improve quality of life [28]. Prior research supports these findings. Xu et al [11] reported that effective communication helps patients correct misconceptions about recurrence and fosters healthier beliefs, thereby reducing FCR. In a controlled trial by Chen et al [12], a self-disclosure intervention significantly increased disclosure levels, reduced negative emotions, and lowered FCR in postoperative patients with breast cancer undergoing chemotherapy. Another study found that enhancing spousal self-disclosure also reduced FCR [13]. This may be due to the fact that individuals with higher levels of self-disclosure receive more external feedback—such as encouragement, experiential guidance, or health-related information—which improves their understanding of the disease and relieves emotional stress. Sharing one’s illness experience and feelings can also enhance emotional support. Therefore, clinical staff should encourage newly diagnosed patients to engage in active and meaningful communication, such as journaling or peer discussion, to express their thoughts and expectations. This practice can enhance self-disclosure, help patients cope with stress, and assist them in making informed treatment decisions.

### Lower Levels of Social Support Are Associated With Higher Levels of FCR

Social support was significantly and negatively correlated with FCR, encompassing both the physical and social or family dimensions. This finding is consistent with the results of Niu et al [14] and Yu et al [15]. A meta-analysis also demonstrated that low levels of social support are associated with increased FCR in patients with breast cancer, while higher levels of support promote positive health perceptions and reduce stress-related recurrence fears [16]. As a protective factor, social support facilitates emotional regulation through interpersonal interaction and helps alleviate symptom distress and fear of recurrence [27]. In the context of FCR, social support acts as a “buffer,” mitigating the negative psychological impact of stressful events [29]. Strong social support not only reduces symptom sensitivity and psychological distress, such as anxiety and depression, but also enhances mental health, decreases FCR, and improves quality of life. Furthermore, it enables patients to approach treatment and recovery with greater positivity and optimism.

### Limitations

This study has several limitations. First, participants were recruited from a single tertiary cancer hospital in Guangzhou using convenience sampling, which may introduce selection bias and limit generalizability. Patients in a specialized urban cancer center may differ from those in community or rural settings in terms of socioeconomic status, access to insurance, and psychosocial resources. Given Guangzhou’s developed health care infrastructure, findings related to financial burden may be influenced by regional insurance policies and economic conditions. In contrast, associations between psychosocial factors (eg, self-disclosure and social support) and FCR may reflect more broadly applicable cognitive and interpersonal processes. Multicenter studies across diverse regions are needed to confirm the generalizability of these findings. Second, the study captured self-disclosure, social support, and FCR at a single time point. Consequently, it lacks longitudinal data to assess how these psychological variables change over time or impact patients across different stages of treatment and disease progression. Third, the cross-sectional design precludes causal inference. Although significant associations were identified between self-disclosure, social support, financial burden, and FCR, the temporal sequence of these variables cannot be determined. Longitudinal or interventional studies are needed to clarify these potential causal pathways. Fourth, sleep quality was assessed using a single self-reported item rather than a validated instrument, which may introduce reporting bias and limit measurement accuracy. Future studies should use standardized measures, such as the Pittsburgh Sleep Quality Index, to provide a more comprehensive assessment.

### Conclusions

Prior to initial treatment, approximately 50% of patients newly diagnosed with breast cancer reported high levels of FCR. Younger age, poorer sleep quality, greater financial burden, lower self-disclosure, and lower perceived social support were associated with higher FCR levels. These findings highlight the importance of early identification of patients at risk for elevated FCR. Although causal relationships cannot be established due to the cross-sectional design, the results provide preliminary evidence to inform future longitudinal and interventional studies aimed at examining whether enhancing psychosocial resources and addressing sleep and financial concerns may help reduce FCR and improve psychological adaptation during treatment decision-making.

## Abbreviations

FCR: fear of cancer recurrence
MOS-SSS-CM: Simplified Chinese Medical Outcomes Study Social Support Survey
